# Activation and Long‐Term Maintenance of Adaptive Immunity in the Central Nervous System: A Double‐Edged Sword?

**DOI:** 10.1002/cns.70697

**Published:** 2025-12-12

**Authors:** Luojinyun Wang, Feng Zhang, Jiehong Wu, Sibo Yang, Daqiang Zhou, Xiaodi Sun, Bohao Chang, Bo Hu, Yifan Zhou

**Affiliations:** ^1^ Department of Neurology Union Hospital, Tongji Medical College, Huazhong University of Science and Technology Wuhan China

**Keywords:** adaptive immunity, antigen presentation, immune surveillance, neuroinflammation, the central nervous system, tissue‐resident memory T cells

## Abstract

**Background:**

For a long time, the brain was considered an organ with “immune privilege”, where microglial cells played a phagocytic role, maintaining immune self‐sufficiency. However, recent studies have revealed the presence of immune‐related structures and immune cell infiltration in the brain, which participates in adaptive immunity.

**Aim of Review:**

This review aims to synthesize recent findings on the activation and long‐term maintenance of adaptive immunity in the central nervous system (CNS), exploring how adaptive immune responses function in pathogen clearance, tumor defense, and CNS inflammation. It highlights both the protective and detrimental roles of adaptive immunity in these contexts.

**Key Scientific Concepts:**

Antigen‐presenting cells (APCs) present antigen information to naive T cells, initiating adaptive immunity in the CNS. Activated T cells can differentiate into effector T cells to perform immediate immune functions or into tissue‐resident memory T cells (TRMs) that persist in the CNS, providing long‐term immune surveillance. Over the past 15 years, studies have shown that adaptive immunity is activated and maintained during intracranial pathogen infections, brain tumors, and CNS inflammation. While adaptive immunity can clear pathogens, eliminate tumor cells, and protect the brain, it can also lead to CNS inflammation under certain conditions, resulting in undesirable outcomes.

## Graphical abstract

A wide spectrum of cells in the brain can engage in antigen presentation, which activates adaptive immunity in the central nervous system (CNS). Tissue‐resident memory T cells (TRM) develop in adaptive immunity in the CNS and provide long‐term immune surveillance, including the clearance of pathogens and tumor cells. Long‐term maintenance of adaptive immunity can also lead to CNS inflammation under certain conditions, resulting in undesirable outcomes.

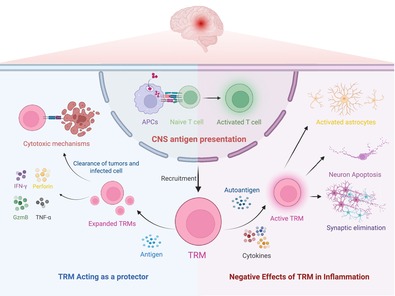

## 1 Introduction

For a long time, the brain has been considered an organ enjoying “immune privilege,” and microglial cells play a phagocytic role and control synaptic pruning, thus maintaining self‐sufficiency in its immune system [1]. In the past two decades, mounting evidence has suggested the presence of immunity‐related structures and immune cell infiltration within the brain, where these cells interact in intricate ways in areas such as the dura mater, choroid plexus, and perivascular spaces [2, 3, 4]. Among these cells, antigen‐presenting cells (APCs) present antigen information to naive T cells, thereby initiating adaptive immunity. Activated T cells have the ability to differentiate into effector T cells to immediately serve immune functions, or into tissue‐resident memory cells (TRMs), which linger in the central nervous system (CNS), providing robust immune surveillance.

Over the past 15 years, studies have demonstrated that adaptive immunity was activated and maintained in various intracranial pathogen infections, brain tumors, and CNS inflammations. Some studies have shown that adaptive immunity can clear pathogens, kill tumor cells, and protect the brain from damage, while others have indicated that, under certain conditions, adaptive immunity can lead to CNS inflammation, resulting in undesirable outcomes. Therefore, investigating the “double‐edged sword” effect of adaptive immunity is crucial for elucidating the mechanisms of inflammatory diseases in the central nervous system and infection‐related long‐term complications. This will subsequently enable the development of precise therapeutic strategies targeting these underlying mechanisms. This review synthesized the studies on the adaptive immune activation and its long‐term maintenance in the CNS, discussed how adaptive immunity works in pathogen clearance and tumor defense, and highlighted recent findings regarding adaptive immunity in CNS inflammation.

## 2 Activation of Adaptive Immunity in the CNS (Figure 1)

**FIGURE 1:**
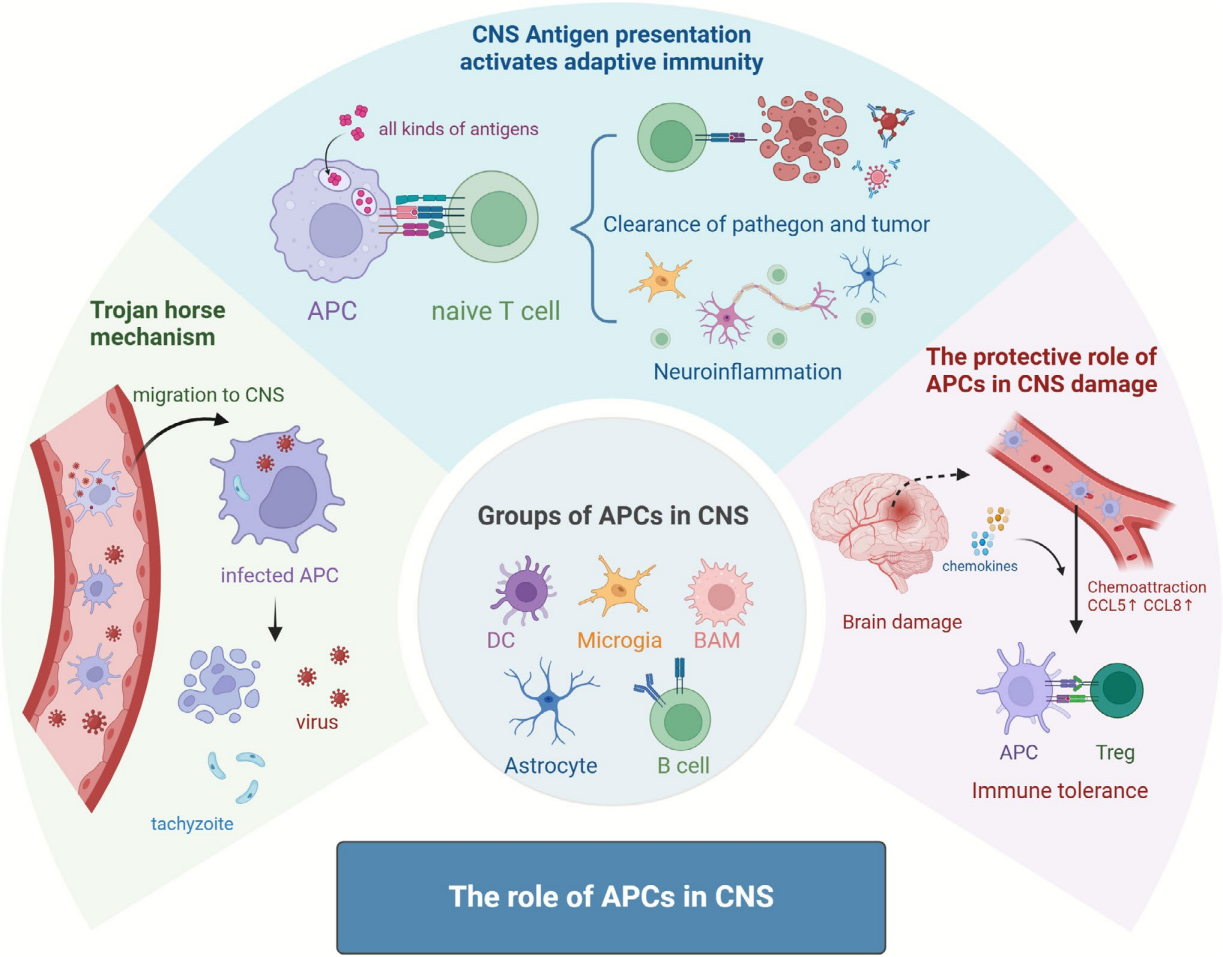
The role of APCs in CNS. A wide spectrum of cells in the brain can engage in antigen presentation, including professional APCs, such as dendritic cells (DCs), resident cells that play unique roles in the brain, such as border‐associated macrophages (BAMs), microglia, and astrocytes. Pathogens can enter APCs and be transported to the CNS, causing intracranial infection. CNS APCs take up various antigens and activate adaptive immunity. This procedure can either promote the clearance of pathogens and tumor cells or lead to neuroinflammation. In brain injuries, such as ischemic stroke, APCs are recruited from peripheral blood vessels into the brain, where they provide protection by promoting immune tolerance.

### 2.1 Subgroups and Localization of CNS Antigen‐Presenting Cells

Antigen presentation plays a pivotal role in the initiation of adaptive immune responses. In the presence of MHC molecules and other co‐stimulatory molecules, APCs provide antigen‐specific signals that activate T cells. This process also takes place in the brain. A wide spectrum of cells in the brain can perform antigen presentation, including professional APCs, such as dendritic cells (DCs), as well as resident cells playing unique roles in the brain, such as border‐associated macrophages (BAMs), microglia, and astrocytes [5]. The antigen‐presenting capacity of these cells in the CNS has been well documented. Goddery et al. isolated cells from the brains of mice infected with intracerebral TMEV and found that myeloid cells in the CNS (including parenchymal microglia and perivascular macrophages) upregulated MHC‐I molecules [6]. They also constructed a CNS myeloid cell MHC‐I knockout model. In this model, upon TMEV infection, infiltration of virus‐specific CD8 T cells was reduced, demonstrating that CNS myeloid cells were implicated in antigen presentation to CD8 T cells [6]. However, another study indicated that in the contexts of viral infections in the central nervous system, immunopathology, and CNS tumor vaccination, the antigen presentation capacity of macrophages is weaker compared to DCs [7]. In a murine model of systemic lipopolysaccharide (LPS)‐induced inflammation, CD3^+^ T cells were detected in the olfactory bulb of both control mice and those administered LPS, 24 h post‐treatment. These T cells were found in close proximity to CD11c‐eYFP‐expressing microglia. Moreover, the sorted olfactory bulb CD11c‐eYFP^+^ and CD11c‐eYFP^+++−^ microglia were capable of inducing antigen‐specific proliferation of naive CD4^+^ T cells in vitro, thereby further confirming the antigen‐presenting function of microglia [8]. Astrocytes, the most abundant cells in the mammalian brain [9], have also been found to be involved in antigen presentation. In chronic active multiple sclerosis (MS) plaques from postmortem patients, GFAP‐labeled astrocytes were positive for MHC II [10].

The anatomical sites of antigen presentation in the CNS are also a topic of significant interest. Despite the CNS's unique architectural features and long‐standing designation as an “immune‐privileged” site, DCs have been found in the meninges, ventricles, choroid plexus, and periventricular organs [11, 12]. Rustenhoven et al. performed a comprehensive immunohistochemical analysis of immune cells involved in adaptive immunity and observed that T cells and MHC II APCs were not uniformly distributed across the dura mater but were highly localized in the peri‐sinus regions [13]. Their study further sorted myeloid cells that captured OVA and found that macrophages and DCs were responsible for most of the OVA uptake and expressed high levels of H_2_, suggesting that the antigen presentation was highly active in these areas [13]. In an experimental autoimmune encephalomyelitis (EAE) mouse model, after intracerebroventricular (i.c.v.) injection of OVA, cells from the leptomeninges, dura mater, and deep cervical lymph nodes (dcLNs) were able to stimulate TOVA cells, whereas cells from inguinal lymph nodes (ingLNs) were less effective in such stimulation. Interestingly, T cell responsiveness was significantly lower in the dura and dcLNs than in the leptomeninges, indicating more antigens were taken up by leptomeningeal APCs. Staining of meninges from MS patients also revealed significant CD8 T cell infiltration, further supporting the notion that the meninges are a crucial site of antigen presentation in the brain [14]. In a systemic LPS‐induced inflammation model, myeloid cells in the choroid plexus and meninges expressed co‐stimulatory molecule CD80, and sorted cells from these regions could activate naïve T cell proliferation in vitro. APCs exhibited stronger T cell activation in the dura mater than in the choroid plexus [8].

### 2.2 CNS Antigen‐Presenting Cells Take Up Various Antigens and Activate Adaptive Immunity

APCs in the CNS are responsible for the presentation of a wide array of antigens and activation of adaptive immune responses. In the CNS, this process provides protection against infection, tumor development, and sterile inflammation. Current research has shown that brain APCs can take up a multitude of pathogen‐derived antigens and present them to T cells. In an experiment with mice infected with recombinant TMEV, mice lacking the Kb LoxP class I molecule (the key molecule for antigen presentation to CD8 T cells) exhibited significantly higher viral loads in the CNS at 7 days post‐infection compared to controls, and brain lymphocyte infiltration was also significantly mitigated, suggesting that the absence of APCs impairs the response of CD8 T cells to viral infection [7]. Similar antigen presentation has been observed in CNS infections with various viruses, such as lymphocytic choriomeningitis virus (LCMV) [15], mouse hepatitis virus (MHV) [16], West Nile virus (WNV) [17], and intranasal vesicular stomatitis virus (VSV) [18]. In addition to viral infections, protozoan infections have also been shown to trigger antigen presentation by APCs. In a *P. berghei* ANKA (a malaria parasite) infection model, CD8 T cell infiltration into the CNS was reduced in Kb LoxP class I‐deficient mice 6 days after infection [7]. Earlier studies on Toxoplasma encephalitis showed that mature DCs isolated from the brains of infected mice could present Toxoplasma antigens to naïve T cells, confirming the ability of brain DCs to present these antigens [19].

In gliomas, APCs can take up tumor antigens and present them through MHC I and II molecules. Friedrich et al. implanted GL261 tumor cells into the brain of mice [20]. On Day 21 post‐inoculation, DCs presenting SIINFEKL, a peptide derived from ovalbumin (OVA), were identified in OVA‐expressing brain tumors, and a high frequency of tumor‐infiltrating SIINFEKL‐reactive CD8^+^ T cells was detected, suggesting that tumor‐associated DCs could functionally present antigens through MHC I [20]. Similarly, in a GL261 glioma model, Kb LoxP class I‐deficient mice had lowered tumor antigen‐specific CD8 T cells compared to the control group and developed more extensive tumor growth over the following month [7]. In a mouse model of glioblastoma (GBM), Bowman‐Kirigin et al. found cDC1 DC subsets infiltrated brain tumors. cDC1‐deficient mice showed reduced T cell activation and did not respond to anti‐PD‐L1 treatment [21]. Flow cytometrical analysis of meningioma and GBM samples from patients identified multiple DC subsets, including cDC1‐equivalent CD141^+^ cDCs, cDC1‐equivalent CD1c^+^ cDCs, and CD14^+^ and CD16^+^ monocytes [21]. Given the role APCs play in tumor antigen presentation and ensuring immune response activation that eliminates tumor cells, therapeutic strategies, such as DC vaccines, have been under investigation. In fact, by re‐applying tumor‐antigen‐loaded DCs to gliomas, strong tumor‐specific T cell responses were induced, which prolonged survival in glioma‐bearing animals [22].

In CNS inflammation, APCs also function as a protector under certain conditions. Grigg et al. identified a population of inflammatory group 3 innate lymphoid cells (ILC3s) that infiltrated the CNS in a mouse model of multiple sclerosis [23]. They found that ILC3s from peripheral tissues did not induce cytokine production by myelin‐specific T cells but induced tolerance instead, as evidenced by a significantly reduced frequency of circulating myelin‐specific T cells in the peripheral blood of ILC3‐tolerized mice [23]. Moreover, ischemic stroke triggers an acute sterile inflammation that involves APCs. Gallizioli et al., by employing a mouse model of transient middle cerebral artery occlusion (tMCAo) stroke, found that infiltrating DCs, including cDC1, cDC2, and plasmacytoid DCs (pDCs), exhibited excellent antigen‐presenting ability [24]. RNA sequencing showed that microglia upregulated Ccl8 and Ccl5, which promoted DC infiltration into ischemic brain tissue, and CCR1 antagonists significantly reduced cDC1 numbers. Depletion of cDC1 in Batf3^−/−^ mice resulted in larger infarcts and more pronounced neurological deficits compared to their wild‐type counterparts [24].

### 2.3 Potential Negative Effects of CNS Adaptive Immune Activation

The migration of and antigen presentation by APCs in the brain is not always protective. In some cases, they may lead to unfavorable outcomes. In certain infections, pathogens can enter APCs and be transported to the CNS, causing intracranial infection through a “Trojan Horse” mechanism. Early studies on Toxoplasma gondii infection in mice showed that CD11c^+^ DCs and CD11b^+^ cells contained tachyzoites, and CFSE staining revealed that these cells migrated from the bloodstream to the brain [25]. Recent studies, by using the *P. berghei* ANKA infection model of experimental cerebral malaria (ECM), also showed that dendritic cells participated in ECM‐related blood–brain barrier disruption. Infected mice specifically depleted of DCs were resistant to ECM, attaining a 100% survival rate, whereas control mice died between days six and seven post‐infection [7]. This “Trojan Horse” mechanism has also been observed in viral infections. Recent studies on SIV‐infected rhesus macaques indicate that CD4^+^ cytotoxic‐like T cells represent a key lymphocyte subset responsible for initiating SIV entry into the brain and triggering neuroinflammatory processes via a “Trojan horse” mechanism [26]. Nipah virus (NiV), which causes encephalitis in humans, was found to infect human primary immature DCs (iDCs) in vitro, and NiV‐infected iDCs showed a threefold increase in migration across endothelial layers, suggesting that DCs act as a pathway for NiV's entry into the CNS [27]. A comparable mechanism was observed in infection with varicella‐zoster virus [28].

In CNS inflammatory diseases, particularly autoimmune lesions, the presentation of self‐antigens by APCs plays a crucial part in the pathological event. In a model of EAE, APC‐mediated antigen presentation and the promotion of inflammation have been extensively observed. In early studies, multiple APC subsets reportedly possessed antigen‐presenting ability in EAE. MHC II^+^ DCs have been found to promote disease progression and CNS inflammation in EAE, with increased DC numbers exacerbating clinical severity in mouse models [29]. Resident microglia or astrocytes in the CNS are thought to function as local APCs to facilitate the recognition of lymphocytes [30]. Certain CD11c^+^ monocyte populations in the CNS have also been found to be implicated in EAE inflammation, forming clusters that target T cells during peak disease phases [31]. In a recent study, Mundt et al. specifically deleted MHC II from CNS cDCs and demonstrated that DCs played a leading role in antigen presentation and the initiation of neuroinflammation in EAE, while microglia and macrophages were dispensable in this process [32].

In summary, the migration of and antigen presentation by APCs in the CNS can lead to pathogen propagation and activation of neuroinflammation, and their roles in pathological conditions warrant careful attention.

## 3 Long‐Term Maintenance of Adaptive Immunity in the CNS

### 3.1 Generation, Residency, and Patrol of TRM

In the local environment of the CNS, the long‐term maintenance of adaptive immunity mainly depends on tissue‐resident memory T cells (TRM). TRM in the brain are initially derived from the circulation. A mouse symbiotic experiment showed that circulating T cells were able to infiltrate the brain and reside there [33]. Within the circulatory system, antigen‐presenting dendritic cells (DCs) are crucial for initiating the differentiation of naive CD8^+^ T cells. DCs provide co‐stimulatory signals and cytokine signals to CD8^+^ T cells and thereby present antigens, pushing T cells into an “effector” state [34]. Subsequently, CD8^+^ T cells differentiate into effector T cells (TE) and memory precursors (MPs). MPs, which express low levels of KLRG1, are inclined to develop into long‐lived memory T cells and have the potential to further differentiate into TCM, TEM, and TRM [35]. An adoptive transfer experiment also demonstrated that downregulated KLRG1 in effector CD8^+^ T cells allowed for their differentiation into TRM [36]. Analysis of TRM also revealed that KLRG1 was downregulated in the brain [37]. In addition to KLRG1 downregulation, TGF‐β secreted by DCs is also critical for the differentiation of TRM. αV integrin^+^ DCs present active TGF‐β to resting naive CD8^+^ T cells, promoting their differentiation into TRM. Mice lacking αV integrin‐expressing DCs exhibited a significant reduction in TRM in epithelial tissues [38]. TGF‐β‐induced CD103 expression also facilitated the tissue residency of CD8^+^ T cells [35].

Upon initial activation and differentiation, CD8^+^ T cells enter tissues under the effect of chemokines. CXCR3 and its ligands CXCL9 and CXCL10 are key chemokine receptor‐ligand pairs that promote the migration of CD8^+^ T cells into tissues. An adoptive transfer experiment examining CXCR3‐deficient T cells showed that the TRM formation in tissues was reduced, while the generation of circulating memory T cells increased [35]. Phenotypic analysis of human T cells isolated post‐mortem from the brain also revealed that TRM in the brain were enriched in the CXCR3 pathway [36]. After TRM enter the brain parenchyma, cytokines and other factors promote CD8^+^ T cell residency, ultimately forming TRM. The persistent presence of antigens helps CD8^+^ T cells express CD103 and reside in the brain. In a mouse model of *Listeria monocytogenes* (LM) infection, when no antigen was present in the brain, no CD103 expression was found, but when there exist OVA antigens, T cells continuously reside in the brain, leading to upregulation of CD103. Additionally, retroviral knockout of CD103 reduced the presence of memory T cells in the brain [39]. CD69, a well‐known marker of TRM, is also highly expressed in brain TRM. In a mouse model of herpes simplex virus (HSV) infection, CD69 expression was found to be essential for the optimal formation of TRM in non‐lymphoid tissues [35]. Induction of CD69 could block sphingosine‐1‐phosphate (S1P) receptor S1P1, thereby preventing S1P‐mediated T cells from exiting lymphoid tissues and enhancing their residency [40]. Phenotypic analysis of peripheral infection‐induced brain TRM revealed that cerebral TRM expressed S1PR1 and encoded the Klf2 gene [41]. The expression of S1PR1 and Klf2 could suppress recirculation, enabling CD8^+^ T cells to stay in tissues [42]. Interleukin‐15 (IL‐15) plays an important role in the long‐term survival of TRM, and IL‐15 responsiveness entails low‐level T‐bet expression and downregulation of eomes [43]. Most CD8^+^ T cells from the brain exhibited moderate T‐bet levels and low eomes levels. CD103 expression was associated with a low‐level eomes expression in brain CD8^+^ T cells, and the highest proportion of cells expressing intermediate‐/low‐level T‐bet was found in the CD69CD103 cell component [36]. In summary, CD8^+^ T cells transform to TRM in the brain under the influence of antigens, various cytokines, and chemokines.

Anatomically, TRM preferentially settle in border regions of the brain, such as perivascular spaces, meninges, and choroid plexus, and patrol these areas. A study used genetically engineered attenuated lymphocytic choriomeningitis virus (rLCMV) to infect mice intracranially and detected TRM at anatomical borders, such as the brain surface structures (meninges and choroid plexus), periventricular regions, and between different brain regions [44]. In mouse experiments inducing peripheral infections to generate brain TRM, live imaging also revealed TRM in the meninges and superficial cortical layers, and they presented some dynamic features, including long‐distance movement within short periods, crawling along the outside of blood vessels, remaining stationary, or moving back and forth. Their average speed was approximately 4 μm/min, indicating a patrolling movement pattern of brain TRM [41]. This is consistent with the recent findings that meningeal lymphatic structures and the perivascular spaces function as immune‐active microenvironments. It is worth noting that the periventricular space is a critical immune‐active compartment between the blood–brain barrier and brain parenchyma. Activated T cells and antigen‐presenting cells localize and interact there, forming an immune‐active environment different from that of the brain parenchyma [45]. An immunohistochemical analysis examined freshly deceased human brains and revealed that CD8^+^ T cells were predominantly located in perivascular spaces, with CD103^+^ T cells being the dominant population. Some CD103^+^ T cells were also found in the brain parenchyma, indicating TRM were distributed in the brain and were capable of migrating into the brain parenchyma [36]. An immunohistochemical study examined the VSV‐infected mouse brains and found that CD103^+^ T cells clustered in the brain parenchyma. Laser capture microdissection of these clusters during early infection and following amplification revealed the presence of viral gRNA, indicating that TRM could migrate to sites of viral infection within the brain parenchyma [39]. A study on T‐cell phenotypes in different anatomical locations of postmortem human brain tissue also revealed that both the borders of the human central nervous system (choroid plexus, leptomeninges) and the intrathecal compartments (cerebrospinal fluid, white matter) contain resident CD8^+^ and CD4^+^ T cells, though phenotypic differences exist. Within the intrathecal compartments, T cells exhibited similar phenotypes, but those in the cerebrospinal fluid showed a higher activation status compared to those in the white matter [46]. Collectively, these findings confirmed that most TRM reside in the meninges, perivascular spaces, and choroid plexus, and can migrate into the brain parenchyma to perform their functions when pathogens invade or inflammation occurs in the brain parenchyma.

Once settling in the brain, TRM can persist over the long term, partially through self‐renewal and partly via recruitment from the circulation. Several experiments have shown that even when the migration of circulating T cells into the brain is blocked or circulating T cells are depleted, brain TRM can still stay for a long time and proliferate upon antigen stimulation [39, 41, 44]. On the other hand, symbiotic experiments in mice have also shown that circulating T cells could infiltrate brain tissue, contributing to the brain TRM pool [33]. Brain TRM are also regulated by PD‐1 and CTLA‐4. PD‐1 is expressed in brain TRM [39] and can protect CD8^+^ T cells from overstimulation, excessive proliferation, and terminal differentiation [47]. CTLA‐4, a homolog of CD28, can block T cell activation [48]. Both CTLA‐4 and PD‐1 likely provide important inhibitory signals to brain CD8^+^ T cells under inflammatory conditions.

### 3.2 TRM Acting as a Protector (Figure 2)

**FIGURE 2:**
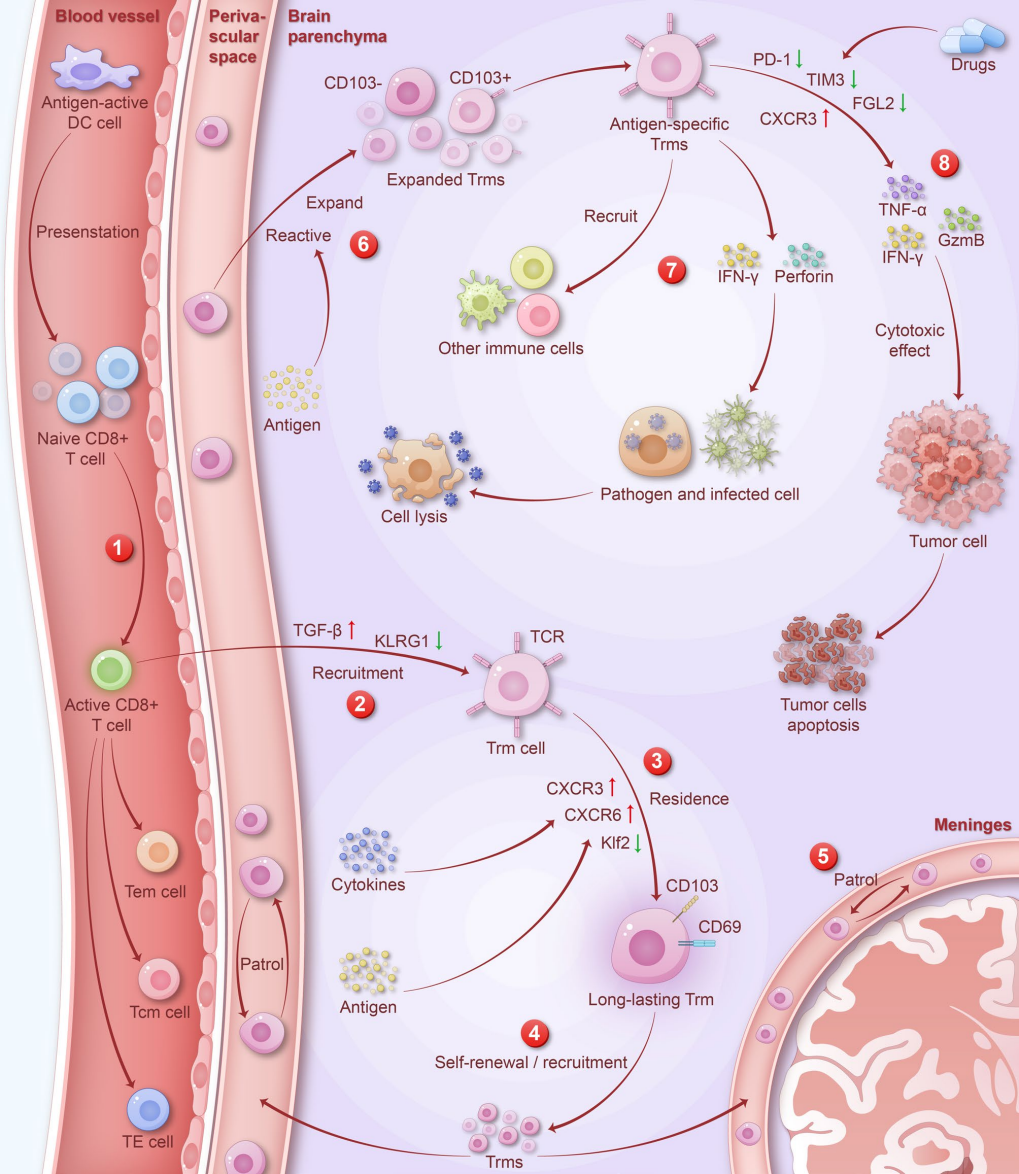
TRM develop, reside, and patrol in the brain to combat pathogens and tumor cells. (1) Dendritic cells (DCs) present antigens to naive CD8^+^ T cells, promoting their differentiation into effector T cells (TE) and memory precursors (MPs). MPs further differentiate into effector memory T cells (TEM), central memory T cells (TCM), and tissue‐resident memory T cells (TRM). (2) The recruitment and differentiation of TRM depend on KLRG1 down‐regulation and TGF‐β secretion by DCs. (3) The long‐term intracranial residence of TRM relies on antigen abundance and the regulation of cytokines, including CXCR3, CXCR6, and Klf2. TRM in the CNS exhibit CD103 and CD69 phenotypes. (4) The renewal of TRM in the brain parenchyma involves both self‐renewal and the recruitment of peripheral T cells. (5) TRM preferentially localize to the perivascular space, meninges, choroid plexus, and other anatomical boundaries, where they actively patrol. (6) Following activation by antigens, TRM rapidly proliferate and differentiate. CD103^−^ TRM are non‐antigen‐specific and rapidly proliferate within 24 h, while CD103^+^ TRM are antigen‐specific, proliferating and differentiating within 15 days. Both types of TRM precede antigen‐specific T cell infiltration. (7) TRM secretes various cytokines to remove pathogens, including IFN‐γ and perforin, which mediate cytotoxicity and rapidly clear pathogens. Additionally, TRM can recruit other immune cells to assist in pathogen clearance. (8) TRM can release lytic particles containing perforin and granzyme B (GZMB) to directly kill tumor cells, or secrete IFN‐γ to inhibit tumor cell growth. The anti‐tumor effect of TRM can be enhanced by inducing TRM production and blocking immune checkpoints, such as PD‐1, to reduce TRM apoptosis.

When the brain experiences various pathogenic infections, brain TRM rapidly proliferate and engage in immune clearance. Current research has found that proliferating TRM can be detected in the brain during infections with different pathogens (Table 1). A recent study demonstrated that physiological microbial exposure in mice can also substantially increase the number of TRM in the brain. Researchers employed a polymicrobial exposure model (involving five BSL‐2 pathogens) to “normalize” the immune systems of specific pathogen‐free (SPF) mice, observing an increase in TRM numbers and their activation. This finding aligns with observations in human autopsies, where TRM acquisition occurs even though the human population may not have experienced overt neurotropic infections, neuroautoimmune diseases, or neurodegenerative disorders throughout their lifetimes [54]. In a mouse model of encephalomyocarditis virus (TMEV) infection, CD69^+^ CD103‐ TRM were observed to proliferate within 24 h, while antigen‐specific CD103^+^ CD69^+^ TRM appeared on Day 15 post‐infection and lingered for up to 60 days or longer. This indicates that brain TRM cells proliferate in a non‐specific manner within 24 h following a viral infection in the central nervous system and undergo further expansion and activation upon antigen exposure [33]. Similarly, in a mouse model of intracranial infection with recombinant lymphocytic choriomeningitis virus (rLCMV), cerebral infection was controlled within 10 days post‐infection, and TRM numbers remained stable 6 weeks later, with about half of the TRM expressing CD103. Regardless of CD103 expression, these cells were able to resist peripheral T cell depletion [44]. Although both CD103^+^ and CD103^−^ TRM play a role in immune responses to infections, CD103^+^ antigen‐specific TRM secrete higher levels of pro‐inflammatory cytokines. A study using a mouse model of *Toxoplasma gondii* infection in the brain found that TNF gene expression was significantly higher in CD103^+^ CD8^+^ T cells than in CD103^−^ CD8^+^ T cells, and the proportion of CD103^+^ T cells producing IFN‐γ and TNF‐α was significantly higher than that of CD103^−^ T cells [55].

**TABLE 1:** Intracranial infection in which TRM were detected.

Pathogen	TRM phenotype	References
HSV	CD69^+^ CD103^+^ CXCR6^+^ PD‐1^+^	[49]
VSV	CD69^+^ CD103^+^ CD122^+^ PD‐1^+^	[39, 41, 50]
MV	CD69^+^ CD103^+^	[51]
LCMV	CD69^+^ CD103^+^ GzmB+bcl‐2^+^	[41, 44]
EBV	CD69^+^ CD103^+^ GzmB+PD‐1^+^	[52]
TMEV	CD69^+^ CD103^+^ CD49a^+^	[33]
LM	CD69^+^ CD103^+^ CD49a^+^ PD‐1^+^	[39, 53]
Toxoplasma	CD103+ Klrg1‐S1PR1‐	

IFN‐γ is a major effector cytokine of TRM and a key player in antiviral responses. Brain TRM have been shown to release IFN‐γ during infections with a wide array of viruses [41, 44]. Additionally, in IFN‐γ knockout (GKO) mice, although brain TRM still proliferated after intracranial viral infection, their ability to clear infected cells and viral RNA was significantly compromised, confirming the importance of IFN‐γ in the antiviral functions of TRM [44]. Brain TRM also directly kill target cells presenting homologous antigens via cytolytic pathways. After rLCMV infection, granzyme B expression was upregulated in brain TRM, and in perforin‐deficient (PKO) mice, there were significantly more infected cells, and viral RNA levels were elevated compared to controls [44]. In a study on multiple sclerosis and Epstein–Barr virus (EBV) infection in the brain, CD103^+^ T cells expressing granzyme B were found in the perivascular infiltrates at the boundaries of chronic active white matter lesions. Immunohistochemical analysis revealed direct contact between EBV‐infected cells and CD69^+^ TRM in the meninges, indicating that brain TRM can recognize homologous viral antigens [52]. These findings suggest that brain TRM can kill pathogens and infected cells through perforin‐ and granzyme‐mediated cytolytic pathways. During infections, TRM secrete various cytokines that can alter the endothelial and local chemokine environment, promoting the recruitment of other immune cells to the tissue. In a study involving recombinant vaccinia virus infection in mice expressing the gp33 peptide, reactivated TRM secreted TNF‐α and promoted dendritic cell (DC) maturation while activating NK cells and enhancing their granzyme B expression [56]. During reinfection, peripheral infection‐induced brain TRM demonstrated an ability to recruit antigen‐specific immune cells from the periphery to control the pathogen [41].

In addition to their role in combating infections, brain TRM are also involved in anti‐tumor immunity. A loss‐of‐function RNA screening identified Runx3 as an important regulatory factor for TRM and suggested that virus‐specific TRM and tumor‐specific TRM share a common transcriptional program that facilitates the establishment of tissue residency [57]. In other human organs, such as the lungs, liver, stomach, uterus, and bladder, the presence of TRM has been found to be positively correlated with more favorable patient prognosis [58]. Studies on tumors in these organs have shown that CD103^+^ T cells could release cytolytic granules containing perforin and granzyme B (GZMB) to directly kill tumor cells, which allows tumor antigens to be used for the activation of new tumor‐specific T cells. Tumor‐specific T cells also produce IFN‐γ, which can inhibit tumor cell growth [59].

Recent studies have also shown that the presence of TRM in the CNS is associated with better prognosis in glioblastoma patients. In experiments using fresh tumor samples harvested from glioblastoma (GB) patients, tumor‐infiltrating immune cells in GB were positively correlated with CD8^+^ CD103^+^ TRM. Blocking PD‐1 or TIM3 on CD8^+^ CD103^+^ T cells enhanced their anti‐tumor activity. Patients with low levels of CD8^+^ CD103^+^ PD1^+^, CD8^+^ CD103^+^ TIM3^+^, and CD8^+^ CD103^+^ PD1^+^ TIM3^+^ TRM subpopulations had significantly better progression‐free survival (PFS) and overall survival (OS) [60]. In experiments targeting FGL2 (an immune‐suppressive regulator) for the treatment of gliomas, blocking FGL2 on T cells increased CXCR3 expression on CD8^+^ TRM, promoted their proliferation, and enhanced granzyme B secretion. These TRM killed tumor cells via cytotoxic mechanisms, thereby curbing glioma progression [61]. Given the anti‐tumor activity of TRM, therapeutic strategies targeting TRM are currently under investigation. Vaccination strategies have been shown to induce TRM [62], while blocking immune checkpoints (such as PD‐1) can reduce TRM apoptosis [60, 61], both enhancing TRM‐mediated local anti‐tumor immunity. In a recent study, researchers developed a novel therapeutic polypeptide vaccine targeting FGL2, whose dominant epitope peptide was tandemly linked to the C‐terminus of the vaccine. Compared to the vaccine without FGL2 linkage, this new vaccine increased the proportion of TRM in the tumor microenvironment of a GBM mouse model and ultimately prolonged the survival of the animals [63]. Although there exists abundant preclinical evidence for taking advantage of TRM in anti‐tumor therapies, their clinical utility still warrants further exploration.

### 3.3 Negative Effects of TRM in Inflammation (Figure 3)

**FIGURE 3:**
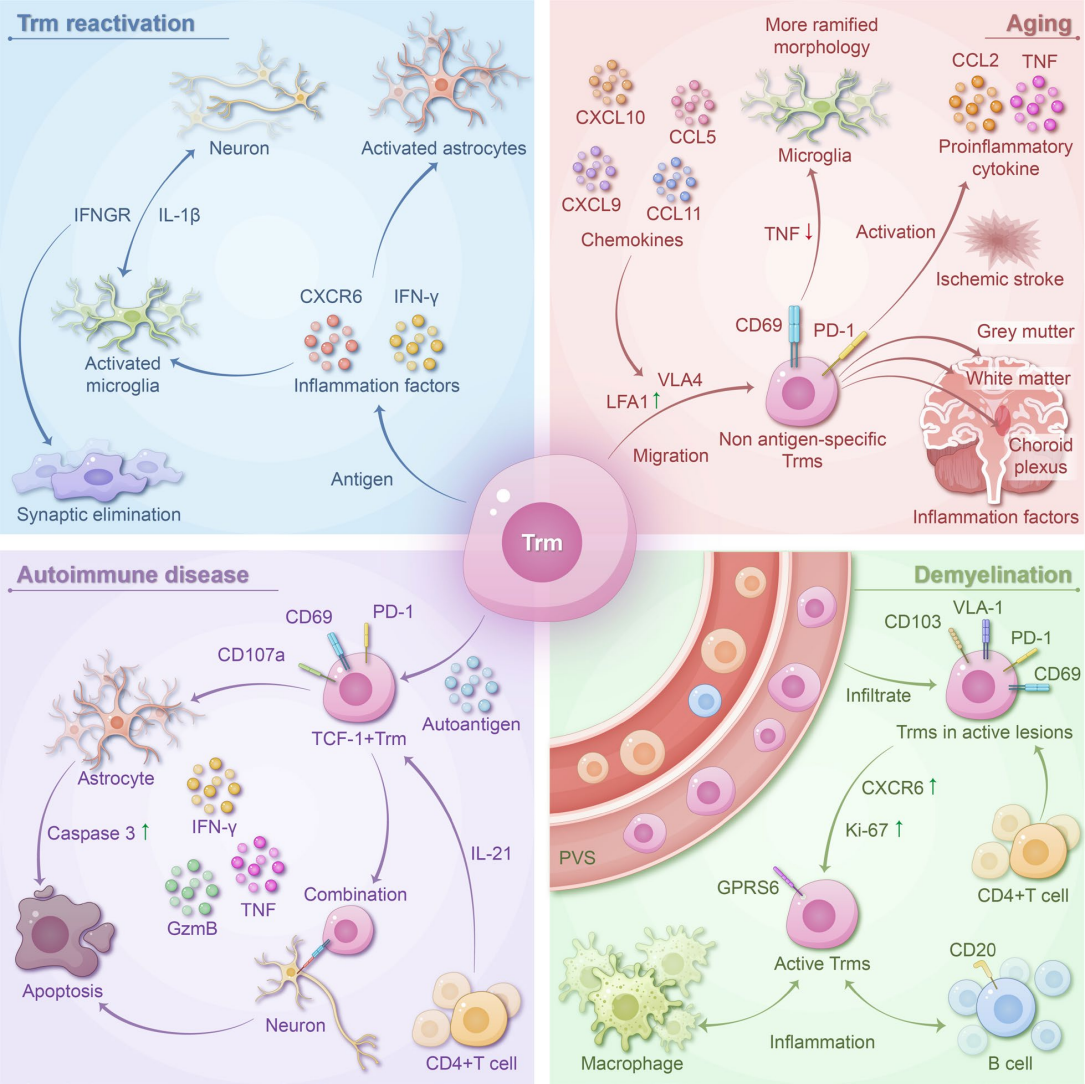
TRM leads to abnormal inflammation in CNS. Following reactivation by pathogen antigens, TRM produce abundant pro‐inflammatory factors, including CXCR6 and IFN‐γ, which activate microglia and astrocytes. Microglia enhance phagocytosis via IFNGR signal transduction and IL‐1β secretion, resulting in synapse elimination. In autoimmune encephalopathy, TRM are activated by autoantigens and CD4^+^ T cells, secreting IFN‐γ, TNF, granzyme B, and perforin, which lead to neuronal loss and astrocyte death. In demyelinating diseases, TRM are recruited and activated in the perivascular space. These cells highly express CXCR6 and Ki‐67 and maintain close contact with macrophages and B cells. In the aging brain, TRM can migrate to the brain in response to various cytokines. These cells are present in the gray matter, white matter, and choroid. Aging brains contain a higher number of CNS‐resident CD8^+^ T cells and have microglia with increased branching in their resting morphology. This is associated with enhanced phagocytosis and decreased TNF. As evidenced in ischemic stroke models, upon stimulation, TRM release pro‐inflammatory cytokines, including TNF and CCL2, which promote the recruitment of peripheral white blood cells.

In the CNS, brain TRM play a protective role against infections and cancer, but they may be detrimental in neuroinflammatory and autoimmune diseases [64]. In patients with CNS demyelinating diseases, such as MS, the level of CD69^+^ CD103^+^ T cells is significantly higher in the cerebrospinal fluid (CSF) than in peripheral blood. Moreover, CD8^+^ TRM‐like cells in the CSF were conspicuously higher in patients with chronic inflammatory diseases, such as MS, Parkinson's disease (PD), and Alzheimer's disease (AD) than in controls (patients with idiopathic normal pressure hydrocephalus) [65]. CD8^+^ TRM‐like cells, with 50% of them expressing CD103, were also detected in lesions of autoimmune encephalitis [66]. These observations suggest that TRM are intimately associated with CNS inflammation.

Reinfection with pathogens can activate brain TRM, which, while fighting infections, may also contribute to CNS inflammation. In mouse models of West Nile virus (WNV) and Zika virus (ZIKV) infections, IFN‐γ secreted by brain TRM and CXCR6 signaling have been shown to activate microglia [67]. Activated microglia, by means of IFNGR signaling and IL‐1 secretion, enhance phagocytosis, leading to synaptic elimination and loss of neurons in the hippocampus, which eventually results in cognitive dysfunction in mice [68]. Similarly, in HIV‐infected mice injected with the HIV‐1 AI9 peptide intracranially, brain TRM proliferated and produced a wealth of pro‐inflammatory cytokines, inducing the upregulation of MHC‐II and PD‐L1 expression on microglia and activation of astrocytes, thereby mediating CNS inflammation [69]. In another experiment in which TMEV‐infected mice were subjected to intracranial injection with homologous peptides, reactivation of brain TRM also led to neuroinflammation, manifested as increased blood–brain barrier permeability and lowered peripheral lymphocyte levels [33].

In autoimmune encephalitis, TRM are also implicated in CNS inflammation. In lesions of neuron‐targeted autoimmune encephalitis, CD8^+^ T cells expressed TRM markers such as Granzyme B, TOX, PD‐1, CD69, and BCL2. In mouse models of autoimmune encephalitis, PD‐1^+^ TRM produced IFN‐γ, TNF, Granzyme B, and perforin, and immunohistochemistry revealed a close interaction between TRM and neurons expressing self‐antigens, leading to neuronal loss and focal neuroinflammation [66]. In another mouse model of autoimmune encephalitis, TRM, when exposed to self‐antigens, proliferated, expressed degranulation markers like CD107a, and co‐produced IFN‐γ and TNF. Histological analysis showed that the proliferating TRM were close to astrocytes, causing astrocyte death [70]. These studies also indicated the crucial role of CD4^+^ T cells in TRM differentiation and maintenance in autoimmune encephalitis. After depletion of CD4^+^ T cells in autoimmune encephalitis mice by using anti‐CD4 antibodies, the number of TRM significantly dropped, and they failed to differentiate into TCF‐1^+^ effector cells, thus losing the capacity for tissue destruction [66, 70]. The latest immune histopathological evaluation on surgical specimens from Rasmussen encephalitis patients reveals that TRM cells increase with ongoing lesion progression. Furthermore, a subset of these TRM cells expressed Granzyme‐B (GrB) and were frequently observed in close proximity to neurons, suggesting their active involvement in neuronal destruction [71].

TRM also participates in inflammatory responses in CNS demyelinating diseases. Flow cytometrical and immunohistochemical examinations of fresh autopsy materials from MS patients revealed that CD8^+^ T cells isolated from lesions exhibited surface markers typical of TRM, including CD69, CD103, PD‐1, VLA‐1, and CD49a [72, 73]. Single‐cell RNA sequencing of CSF cells from MS patients also found a phenotypically diverse population of CD8^+^ TRM [74]. These cells are primarily located in perivascular spaces [70, 72, 73], but in active lesions, the proportion of CD8^+^ T cells infiltrating the brain parenchyma is elevated. In the chronic phase of EAE model mice, enrichment of CD4^+^ TRM with a pro‐inflammatory phenotype was observed, and it was found that CD4^+^ TRM accumulates in inflammatory lesions in the brains of individuals with progressive MS. Depleting TRM cells can decrease disease severity during the chronic phase of EAE [75]. TRM‐like cells have also been found in postmortem or biopsy tissues from patients with NMOSD [70, 76]. In the IA lesions of NMOSD, CD103^+^ CD3^+^ TRM had higher levels of aryl hydrocarbon receptor (AHR), while in I/EA lesions of NMOSD, GZMB^+^/PRF1^+^ CD103^+^ TRM were prominent, indicating the potential of TRM to induce inflammation in NMOSD lesions [76]. Although TRM have been observed in patients with MS and those with NMOSD presenting signs of activation, the cytotoxic effects of TRM in CNS demyelinating diseases have not been fully confirmed and require further investigation.

As the global population ages, aging and neurodegenerative diseases have become an area of active investigation. In the brains of aged mice, CD45^+^ leukocytes were flow cytometrically found to be significantly increased, being virtually three‐fold higher than in younger mice, with T cells comprising a large proportion of these cells. Moreover, cytokine assays in aged mouse brains also showed significant upregulation of CCL5 (RANTES), CCL11 (Eotaxin), CXCL9 (IFN‐γ‐induced factor), and CXCL10 (IFN‐γ‐inducible protein), which are essential for the migration of activated T cells [77]. These results suggest that the aging brain is more permissive to the entry of lymphocytes. Furthermore, multiple studies have found that CD8^+^ TRM accumulated in the brains of aged mice. Flow cytometrical, transcriptomic, and immunohistochemical analyses have all revealed the presence of TRM in aged mouse brains, which were CD69^+^ CD103^+^ PD‐1^+^ cells, with CXCR6 upregulated and CXCR7 downregulated, and TRM accumulation increasing with age [33, 77, 78]. Correlation analysis showed that aged brains with a higher number of CNS‐resident CD8^+^ T cells had more microglia with a more branched resting morphology, and with phagocytosis enhanced and TNF production reduced. Aged brain TRM, when stimulated (e.g., in an ischemic stroke model), released pro‐inflammatory cytokines, such as TNF and CCL2, which promoted the recruitment of peripheral leukocytes [77]. TRM infiltration has also been observed in neurodegenerative diseases. In CSF of AD patients, clonally expanded and antigen‐specific CD8^+^ T cells were detected [79], and flow cytometry and immunohistochemistry in APP‐PS1 mice revealed CD8^+^ TRM, with GO enrichment and KEGG pathway analysis showing that genes related to the type I IFN signaling pathway, such as Ifnar2 and Irf9, were upregulated [78]. Blocking type I IFN signaling improved age‐related neuroinflammation and behavioral deficits [80]. Recent studies on AD have revealed that CD8^+^ TRM accumulate in the brains of AD mice, producing granzymes and inducing neurofunctional impairment through the GrK–PAR‐1 axis. The activation of neuronal PAR‐1 by GrK in both mice and humans leads to hyperphosphorylation of tau protein in vitro, suggesting that CD8^+^ T cells can directly sustain tau pathology during AD [81]. Postmortem immunohistochemistry of the substantia nigra (SNpc) from Parkinson's disease patients demonstrated that practically all CD8^+^ T cells were CD69^+^, with half being CD103^+^, indicating TRM infiltration in Parkinson's disease lesions. This study also found that half of the CD8^+^ T cells expressed IFN‐γ, and immunofluorescence imaging revealed granzyme B (GrzB) granules within CD8^+^ T cells, suggesting that the TRM in Parkinson's lesions might be potentially cytotoxic [82].

## 4 Conclusion and Prospect

As discussed earlier, the process of antigen presentation activates adaptive immunity in the brain, with TRM cells providing long‐term adaptive immune protection against infections after the acute phase. Over the past decade, the critical and extensive roles of both adaptive immunity and TRM cells in infections, cancer, and neuroinflammation have been well documented. Given their protective effects, the activation and long‐term maintenance of adaptive immunity have become attractive targets for current vaccine development. Vaccines currently under investigation often employ a “Prime and Pull” strategy, in which vaccination activates the immune system, and then cytokines are locally administered to enhance the function of TRM cells within tissues [83]. For example, in herpes simplex virus infections, vaccines using this strategy have been applied to mice and guinea pigs, resulting in a significantly lower recurrence rate compared to vaccination alone [84]. With CNS tumors, vaccines prepared by exposing ex vivo DCs to tumor antigens have been shown to elicit effective and durable immune responses against tumors [85]. The application prospect of targeting TRM cells is broad, including methods to increase TRM cell numbers within solid tumors using therapeutic vaccines [86], and PD‐L1 blockade to reduce TRM cell apoptosis, which has been demonstrated in several animal studies aimed at controlling tumor progression.

In CNS inflammation, APCs take up self‐antigens and activate inflammation, while TRM cells, via the CXCR6 pathway, activate other immune cells and release pro‐inflammatory factors, such as IFN‐γ, TNF, Granzyme B, and perforin, which exacerbate inflammation. These negative effects have been widely observed. However, most experiments come from established animal models (e.g., the EAE model), and the initiating factors for adaptive immune activation in human CNS inflammatory diseases remain poorly understood. However, adaptive immune activation and maintenance do not always exert negative effects in all brain inflammatory responses. For instance, in the sterile inflammation of ischemic stroke, cDC1 cells have been shown to be conducive to the amelioration of brain ischemia. In Alzheimer's disease, CD8^+^ T cells that reside in the brain have been found to restrict β‐amyloid deposition and slow cognitive decline [86]. Studies in rLCMV‐infected mice also demonstrated that brain TRM cells protected the CNS against lethal immune pathology in an antigen‐dependent manner [44].

Given the current focus on the negative effects of adaptive immune activation and long‐term maintenance in inflammation, treatments targeting this process are still believed to be risky. Therefore, in the future, understanding the initiating factors of adaptive immune activation in inflammation and controlling associated risks will be crucial to harnessing this “double‐edged sword” for human benefits.

## Author Contributions

Luojinyun Wang, Feng Zhang, and Jiehong Wu contributed equally to this work. Yifan Zhou and Bo Hu revised the manuscript and approved the final manuscript.

## Funding

This work was supported by the grants from the National Natural Science Foundation of China (No. 82371341 to Y.Z., No. 82330040 and 82090044 to B.H.), Young Elite Scientists Sponsorship Program by CAST (No. 2022QNRC001), Noncommunicable Chronic Diseases–National Science and Technology Major Project (grant 2024ZD0527901 to B.H), and National Key Research and Development Program of China (grant 2024YFC3044800 to B.H).

## Ethics Statement

The authors have nothing to report.

## Consent

All authors have been involved in writing the manuscript and consented to publication.

## Conflicts of Interest

The authors declare no conflicts of interest.

## Data Availability

Data sharing not applicable to this article as no datasets were generated or analyzed during the current study.
